# *Borrelia miyamotoi*: 43 Cases Diagnosed in France by Real-Time PCR in Patients With Persistent Polymorphic Signs and Symptoms

**DOI:** 10.3389/fmed.2020.00055

**Published:** 2020-02-28

**Authors:** Michel Franck, Raouf Ghozzi, Julie Pajaud, Nadou E. Lawson-Hogban, Marie Mas, Alexis Lacout, Christian Perronne

**Affiliations:** ^1^ADNucleis, Grézieu la Varenne, France; ^2^Hôpital de Lannemezan, Service Infectiologie, Fédération Française contre les Maladies Vectorielles à Tiques, Lannemezan, France; ^3^Clinique Convert, Médecine Générale, Service des Urgences, Bourg en Bresse, France; ^4^Centre de diagnostic ELSAN, Centre Médico – Chirurgical, Aurillac, France; ^5^Hôpital Universitaire Raymond Poincaré (Assistance Publique – Hôpitaux de Paris), Département d'Infectiologie, Université de Versailles – Saint Quentin, Paris-Saclay, Garches, France

**Keywords:** *Borrelia*, *Borrelia miyamotoi*, real-time PCR, borreliosis, Lyme disease, relapsing fever, post-treatment Lyme disease syndrome

## Abstract

**Background:** *Borrelia* species are divided into three groups depending on the induced disease and the tick vector. *Borrelia miyamotoi* is a relapsing fever *Borrelia* but can induce symptoms related to Lyme disease. Discovered in 1995, it is found in ticks around the world. In France, this species of *Borrelia* has been isolated in ticks and rodents, but was not yet observed in humans.

**Objective:** The aim of the study was to look for *B. miyamotoi* in symptomatic patients.

**Methods:** Real-time PCR was performed on 824 blood samples from patients presenting symptoms of persistent polymorphic syndrome possibly due to tick bite, a syndrome recognized by the French Authority for Health, which is close to the post-treatment Lyme disease syndrome. PCR was also performed on 24 healthy control persons. The primers were specifically designed for this particular species of *Borrelia*. The sequence of interest of 94 bp is located on the *glpQ* gene. Sequencing of amplification products, randomly chosen, confirmed the amplification specificity. To better investigate cases, a clinical questionnaire was sent to the patients PCR-positive for *B. miyamotoi* and to their physician.

**Results:** This search revealed a positive PCR for *B. miyamotoi* in the blood from 43 patients out of 824 (5.22%). PCR was negative in all control persons. A clinical chart was obtained from 31 of the 43 patients. A history of erythema migrans was reported in five of these 31 patients (16%). All patients complained about fatigue, joint pain and neuro-cognitive disorders. Some patients complained about respiratory problems (chest tightness and/or lack of air in 41.9%). Episodes of relapsing fever were reported by 11 of the 31 patients (35.5%). Chilliness, hot flushes and/or sweats were reported by around half of the patients. *B. miyamotoi* may not cross-react with *B. burgdorferi* serology.

**Conclusion:** This study is the first to detect *B. miyamotoi* in human blood in France. This series of human *B. miyamotoi* infection is the largest in patients with long term persistent syndrome. Our data suggest that this infection may be persistent, even on the long term.

## Introduction

Spirochetes of the genus *Borrelia* are divided into three major groups according to the vector and/or the pathology they can cause. Bacteria of the first group such as *B. duttonii* and *B. hermsii* are responsible for relapsing fevers, transmitted by soft ticks (*Argasidae*). Bacteria of the second group, such as *B. burgdorferi* and *B. afzelii* are the agents of Lyme disease, transmitted by hard ticks (*Ixodidae*). The third group includes species phylogenetically close to species of the first group, but transmitted by hard ticks, including *B. theileri* affecting cattle, *B. lonestari* affecting deer and *B. miyamotoi* affecting rodents (*Apodemus argenteus, Apodemus flavicollis, Myodes glareolus, Peromyscus leucopus*) and birds (*Cardinalis cardinalis, Parus major, Carduelis chloris*), which serve as intermediate reservoirs before humans (1–3).

Lyme disease, or Lyme borreliosis, is the most common tick-borne disease in the northern hemisphere. In Europe, the bacteria belonging to the complex *Borrelia burgdorferi sensu lato* (*B. burgdorferi s.l*.) are transmitted by the ticks of the genus *Ixodes*. The geographical distribution of Lyme disease is linked to that of the vector, mostly found in cool and humid habitats, such as forests. In France, the incidence of the disease varies according to the region studied, increases with years and is now observed on the whole mainland territory with an incidence of 104 cases per 100,000 inhabitants in 2018 (4, 5). However, the lack of physicians' obligation to report cases of Lyme disease makes difficult to determine its precise incidence and location. Furthermore the tick bite is often unnoticed by the patient. The primary stage of the disease is characterized by erythema migrans, a specific sign but not constant. Patients presenting with later stage of Lyme disease suffer from subjective or non-specific polymorphic signs and symptoms which may persist after the end of currently recommended antibiotic treatments. In most of the cases, there is asthenia, possibly disabling, with pain which may be localized in joints, muscles, bones or of neurologic origin. Pain is often migrating. Many patients complain about neurocognitive disorders. Most of the patients present with objective signs from different organs or systems (neurologic, rheumatologic, cutaneous, cardiac, visual…) but these signs are not specific and may be observed in other diseases. Lyme disease serology may be negative (6). Thus, physicians lack accurate diagnostic tests to better investigate the possible causes of these nonspecific syndromes. To further complicate the issue, it has been shown that some of these patients may suffer from other co-infections due to bacteria or parasites such as *Babesia*. Different names have been proposed to define these signs and symptoms, often mentioned as “post-treatment Lyme disease syndrome,” PTLDS (7). In France, the denomination recognized by the High Authority for Health (Haute Autorité de Santé, HAS) in the official French Recommendation of Good Practice (June 2018) is “persistent polymorphic syndrome possibly due to a tick bite,” (SPPT) (8). The difference between SPPT and PTLDS is that a diagnosis of Lyme disease has not to be proven and patients may have not been treated. It is now established that various species of *Borrelia* may be isolated from humans. *Borrelia miyamotoi*, discovered more than two decades ago, has been isolated from ticks and from patients in various regions of the world. Its real incidence in populations is not yet established.

*B. miyamotoi* was first described in 1995, isolated from ticks of the genus *Ixodes persulcatus* (9). Later, it was also observed in other tick species such as *I. scapularis, I. Pacificus*, and *I. ricinus* (10). Its DNA has shown similarities with other *Borrelia* species. It was named *Borrelia miyamotoi* sp. nov. (reference strain: HT31) and has been first classified with the *Borrelia* involved in relapsing fevers (9). However, further studies have shown that *B. miyamotoi* could provide in some patients signs and symptoms similar to Lyme disease. In 2011, a Russian team highlighted for the first time the presence of *B. miyamotoi* in humans. A large proportion of patients showed signs and symptoms similar to those caused by *B. burgdorferi s.l*., including fever, headache, myalgia and arthralgia (11). The authors also found a high incidence of *B. miyamotoi* in the study area. Infections with *B. miyamotoi* seemed more severe than those observed with *B. burgdorferi* or *B. garinii*. In a study by Lee et al. (12), a highly conserved 357-bp segment of 16S rDNA gene of *B. burgdorferi s.l*. plus the correspondent 358 bp-segment of *B. miyamotoi* were amplified by nested PCR (single pair of primers). Amplicons were used as templates for direct Sanger DNA sequencing. This technique allowed, in winter, to detect spirochetemia in 14 patients. Among these, the bacterium involved was *B. miyamotoi* in four cases and a combinaison of *B. miyamotoi* and *B. burgdorferi* in one case. In immunocompromised patients, *B. miyamotoi* infection caused meningoencephalitis in the United States and in Europe in the Netherlands (13, 14). In France, the first study on *B. miyamotoi* was carried out in 2014 on ticks and rodents (15), demonstrating that 3% of the ticks and 5.55% of the rodents were infected with *B. miyamotoi*. Strain sequencing showed the same genotype not only in ticks, rodents but also in one Dutch patient reported by Hovius et al. (14). In Japan the same year, two publications showed that *B. miyamotoi* could be present in patients presenting with signs and symptoms suggesting Lyme disease (16, 17). Subsequently, *B. miyamotoi* has also been detected in other European countries such as Belgium and England (18, 19). In a study conducted in New York state using multiplex real-time PCR on 796 clinical specimens (blood and CSF), *B. miyamotoi* was found in eight cases (20). The frequency of *B. miyamotoi*, as a human pathogen, as well as the severity of some related signs and symptoms such as meningoencephalitis, make prevention, diagnosis and treatment of this infection essential (21). Furthermore, *B. Miyamotoi* may be resistant to some antibiotics such as amoxicillin, at least *in vitro* and has the ability to bypass the body's immune mechanisms, such as the complement by means of a surface protein, a factor H-binding protein, termed CbiA (complement binding and inhibitory protein A) (22–24). The local immune response is influenced by the tick, which secretes a multitude of immunosuppressive salivary factors that target the organism defense molecules. The subsequent immune reaction is delayed or incomplete thanks to the intervention of glycoproteins, called “evasins,” which will bind to the chemokines secreted by the host, inhibiting their actions (25). A known problem of infections caused by (some, if needed) strains of group *Borrelia s.l*. is the reappearanceor persistence of signs and symptoms after a classical treatment (26, 27). Recently, a study conducted in Russia, confirmed the presence of *B. miyamotoi* in 70 of 473 patients at the early stage of signs and symptoms occurring after a tick bite (28). This study showed that the median time for detection of *B. miyamotoi* in blood was 4 days after inoculation. No human case of *B. miyamotoi* has been described in France yet.

The purpose of this study, carried out in a population different from that studied in 2018 by Karan et al. (28), was to look for *B. miyamotoi* in the blood of patients living in France and suffering from a persistent polymorphic syndrome possibly due to a tick bite (7, 8). In case of positive tests, we obtained a first approximation of the incidence of the infection. Due to numerous positive tests, the study was further completed with a clinical evaluation. A standardized questionnaire was sent to the patients detected positive and to their physicians to obtain information about their medical history and clinical presentation.

## Patients and Methods

### Patients and Samples

Blood samples were drawn from two groups of people. A control group was made up of healthy students of the University of Angers, not expressing signs or symptoms and located in a rural region of France (*n* = 24). The second group included patients, expressing signs and symptoms compatible with a persistent polymorphic syndrome possibly due to a tick bite and living in different regions of France (*n* = 824). These signs and symptoms included a range of conditions associated with fatigue, sleep disturbance, neurological/musculoskeletal pain, and cognitive dysfunction, lasting for at least 6 months. A questionnaire was used, including the main signs and symptoms usually observed during SPPT/PTLDS (7, 8).

Five milliliters of blood were collected by venous puncture in tubes with EDTA as anti-coagulant, before any antibiotic treatment and were sent in Vacutainer® K2 tubes.

### Selection of Primers

**Figure 1:**
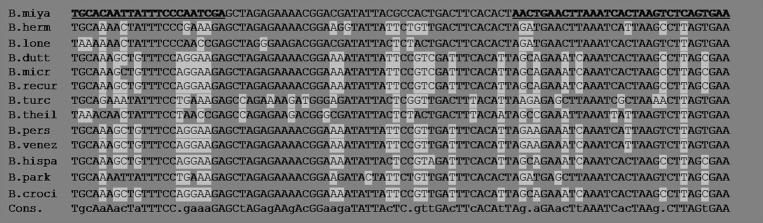
Several sequences of the *glpQ* gene portion used for the detection of *B. miyamotoi* and belonging to other species of the recurrent fever group of *Borreliae* are aligned and compared. Bold and underlined: primers for the target *B. miyamotoi*. The *glpQ* gene, in the current state of the art and genome annotations has not been described in *Borrelia* species other than those of the recurrent fever group. Highlighted: the sequence differences of some recurrent fever *Borrelia* compared to that of *B. miyamotoi*. The following sequences are aligned: *B. miyamotoi* (D43777.1), *B. hermsii* (DQ855539.1), *B. lonestari* (AY368275.1), *B. duttoni* (DQ346787.1), *B. microti* (EU914144.1), *B. recurrentis* (DQ346781) .1), *B. turcica* (AB529430.1), *B. theileri* (KF569938.1)*, B. persica* (AY530742.1)*, B. venezuelensis (*MG651651.1)*, B. hispanica* (GU357572.1)*, B. parkeri* (MH704900.1)*, B. crocidurae* (JX292940.1). The regions of the primers are very different between species.

To allow the detection of *B. miyamotoi*, primers targeting the gene *glpQ* (Accession KU845211.1) of *B. miyamotoi* and framing of 94 bp portion of gene were used (29) (Table 1, Figure 1). The primers used in this study were derived from an existing publication by Reiter et al. who developed a new PCR approach for the detection of *B. miyamotoi* in ticks (30). Alignment of the sequence of interest of *B. miyamotoi* with the same portions of sequences in the genome of other *Borrelia* species confirms the specificity of the primers (Figure 1). In order to be more sensitive, a PCR simplex kit specific for *B. miyamotoi* was used, to avoid the loss of sensitivity common with multiplex kits.

**Table 1:** Sequences of the primers used for *B. miyamotoi* PCR and sequences of housekeeping gene (GAPDH) primers.

**Target**	**Gene**	**Primers**	**Probes**
*Borrelia miyamotoi*	Glycerophosphodiester phosphodiesterase *glpQ*	F 5′ TGCACAATTATTTCCCAATCGA 3′ R 5′ TTCACTGAGACTTAGTGATTTAAGTTCAGTT 3′	
Human	*GAPDH*	F 5′ GAAGGTGAAGGTCGGAGT 3′ R 5′ GAAGATGGTGATGGGATTTC 3′	5′-6-FAM CAAGCTTCCCGTTCTCAGCC-BHQ1-3′

### Robustness of PCR Mixes

The portion of the *glpQ* sequence of *B. miyamotoi* was synthesized and introduced into a plasmid to obtain a control DNA and facilitate its multiplication. This control DNA was used to validate the amplification mix. Serial dilution of the plasmid was performed and amplified to determine the robustness parameters of the *B. miyamotoi* PCR kit: the limit of detection (LOD), the limit of quantification (LOQ), the repeatability and the reproducibility (Table 2).

**Table 2:** Characteristics of the *B. miyamotoi* PCR kit.

**PCR Mix**			**LOD**		**LOQ**		**Repeatability**	**Reproducibility**
	**Tm**	**Mean efficacy**	**GU/PCR**	**GU/ml**	**GU/PCR**	**GU/ml**	**Mean CV**	**Mean CV**
*Borrelia miyamotoi*	79.5°C	106.3%	12.5	1,041	18.8	1,567	0.85	1.22

*Tm, melting temperature; LOD, limit of detection; LOQ, limit of quantification; GU, genome unit; CV, coefficient of variation*.

### DNA Extraction and Purification

The DNA was extracted without any prior treatment using 300 μl of whole blood with an equal volume of ADNucleis extraction buffer (5 M guanidium thiocyanate, 500 mM TrisHCL, 50 mM EDTA, 20% Tween 20, 20% Triton X-100, 750 μg proteinase K). After incubation for 20 min at 56°C and 15 min at 80°C, the extracted DNA was purified by means of silica magnetic beads and eluted in 250 μl of elution buffer (10 mM TrisHCl, pH 8.5).

### Control of the Extraction

Glyceraldehyde-3-phosphate dehydrogenase (GAPDH) was used as a housekeeping gene as an internal control for PCR extraction and inhibition. The extracted samples were first checked with a PCR targeting the GAPDH gene. If the results of this PCR were consistent (Ct of GAPDH below 32), the samples were then analyzed for the other pathogens. The sequence of interest of GAPDH was inserted into a plasmid to be the *B. miyamotoi* target and this plasmid was used as a positive DNA for the validation of GAPDH primers and PCR mix as well as a positive control for subsequent PCRs. The primers used for GAPDH are described in Table 1.

### Real-Time PCR

Real-time PCR was carried out in a total volume of 50 μl with a PCR mix containing ADNucleis PCR buffer (20 mM Tris-HCl, 10 mM NH_4_SO_4_, 10 mM KCl, 2 mM Mg2+, 0.1% TritonX-100, pH 8.8), 2 mM of each dNTP, 600 nM of each primer, 1 μl of Evagreen and 5 units of *Taq* polymerase ADNucleis. Twelve μl of extracted samples were amplified.

An initial denaturation step of 5 min at 95°C was followed by 42 cycles of 15 s at 95°C and 40 s at 60°C (hybridization-elongation). The dissociation curves were generated by a last step of 10 min with temperature increments from 75 to 95°C.

### Quantification

Positive samples were quantified using a standard curve obtained by amplifying known and calibrated concentrations of control DNA of the desired targets. Quantification was obtained using the standard curve equation (Ct = a (Log10 [DNA]) + b) where “a” is the slope and “b” the intercept of the curve. The results were expressed in genome units (UG) per ml of blood.

### Sequencing

The PCR results of some samples were verified by sequencing. A positive sample at the first PCR was amplified again in a second PCR with the same mix and primers. The product of this second PCR was then sent to an external provider for the sequencing of the obtained amplicons. Primers were supplied to the provider. The sequences obtained after sequencing were then compared to the expected sequence of the amplicon, which is specific of the target.

## Results

### Research of the Presence of *B. miyamotoi* by qPCR on Healthy Control Volunteers

The presence of *B. miyamotoi* was searched by qPCR on the control group of 24 healthy asymptomatic students. For all extracted blood samples, a Ct of less than 32 was detected for the GAPDH extraction control, which allowed further investigation. The results showed that none had *B. miyamotoi* infection (Table 3).

**Table 3:** Lack of detection of *B. miyamotoi* in the healthy persons of the control group.

	**PCR inhibition**	**Ct GAPDH values**	**Detection**
FDC071	No	29.44	Not detected
MCM072	No	24.46	Not detected
MGA073	No	28.57	Not detected
MFA074	No	28.47	Not detected
FBF075	No	27.7	Not detected
MDW076	No	27.76	Not detected
FDT077	No	30.97	Not detected
MAJ078	No	28.29	Not detected
MMC079	No	28.81	Not detected
FMS081	No	28.08	Not detected
MSL082	No	31.28	Not detected
MMD085	No	31.55	Not detected
MPA088	No	30.57	Not detected
FVA089	No	29.98	Not detected
MGW092	No	31.17	Not detected
FDN093	No	29.15	Not detected
MBA094	No	31.87	Not detected
FFS095	No	28.32	Not detected
FBA096	No	28.7	Not detected
FGA098	No	28.89	Not detected
MACA101	No	26.54	Not detected
MLS103	No	30.83	Not detected
FLH105	No	31.77	Not detected
FLL106	No	28.44	Not detected
Positive control	No	22.83	Detected
Negative control	No	0	Not detected

*B. miyamotoi was searched by qPCR on a control group of 24 healthy asymptomatic students. All analyzed bloods were negative*.

### Results of Analyses on Symptomatic Patients

**Figure 2:**
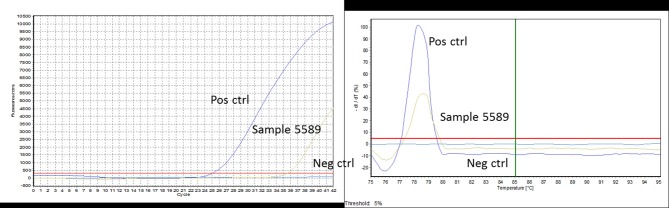
An example of PCR curves obtained for sample 5589. The positive control well shows a Ct of 25 with a specific Tm of 79°C. Sample 5589 is amplified with a Ct value of 35 and the same specific Tm of 79°C. The negative control well shows no Ct and no Tm.

After the confirmation of the absence of *B. miyamotoi* in the group of healthy people, analyses were performed on the second group of symptomatic patients. Out of a total of 824 analyses, 43 samples were detected positive by qPCR for *B. Miyamotoi* (Figure 2), which corresponds to 5.22% of the patients. Of these 43 samples, *B. miyamotoi* could be quantified in 21 cases. In the remaining 22 cases, *B. miyamotoi* concentration was below the limit of quantification (Table 4).

**Table 4:** Results and quantification of samples detected positive for *B. Miyamotoi*.

**N**	**ADNucleis ID**	**PCR results**	**Ct**	**Tm (^**°**^C)**	**Quantification GU/PCR**	**Quantification GU/ml**	**Comments**
5	6107	Detected (LOD)	36.87	78.3	NA	NA	Detected but not quantifiable
7	5557	Detected (LOD)	36.74	78.6	NA	NA	Detected but not quantifiable
8	5113	Detected (LOD)	35.73	78.6	NA	NA	Detected but not quantifiable
9	5914	Detected	34.76	78.8	4.0E+01	2.79E+03	–
10	6072	Detected (LOD)	39.4	79	NA	NA	Detected but not quantifiable
11	6129	Detected (LOD)	39.39	79	NA	NA	Detected but not quantifiable
12	6273	Detected (LOD)	36.91	78.4	NA	NA	Detected but not quantifiable
13	6591	Detected	25.92	79	2.4E+04	1.68E+06	–
14	6594	Detected	31.59	78.6	1.5E+04	1.07E+06	–
15	6864	Detected (LOD)	33.82	78.2	1.99E+00	1.38E+02	Detected but not quantifiable
16	6784	Detected (LOD)	32.85	78.3	4.08E+00	2.83E+02	Detected but not quantifiable
17	7086	Detected (LOD)	37.22	78.5	2.15E+01	1.49E+03	Detected but not quantifiable
18	6527	Detected	34.26	79.5	2.7E+03	1.89E+05	
19	6749	Detected	29.91	79.7	4.58E+04	3.18E+06	–
20	6213	Detected (LOD)	39.66	79	NA	NA	Detected but not quantifiable
21	6630	Detected (LOD)	38.19	78.5	1.13E+01	7.88E+02	Detected but not quantifiable
22	6362	Detected	34.41	79.5	2.5E+03	1.71E+05	–
23	5815	Detected	30.13	79.1	4.0E+04	2.76E+06	–
24	6585	Detected	34.9	79	1.8E+03	1.25E+05	–
25	7147	Detected	36.49	78.2	9.47E+02	6.58E+04	–
26	6136	Detected (LOD)	37.52	78.4	NA	NA	Detected but not quantifiable
36	6235	Detected (LOD)	39.02	79.5	NA	NA	Detected but not quantifiable
37	6228	Detected (LOD)	35.76	79	NA	NA	Detected but not quantifiable
38	6231	Detected (LOD)	36.56	79	NA	NA	Detected but not quantifiable
39	6301	Detected (LOD)	38.53	79	NA	NA	Detected but not quantifiable
40	6407	Detected (LOD)	38.91	78.7	NA	NA	Detected but not quantifiable
41	6596	Detected	34.01	79.5	3.2E+03	2.22E+05	–
42	5589	Detected	34.13	79.5	3.0E+03	2.06E+05	–
43	6600	Detected	34.53	79.5	2.3E+03	1.59E+05	–
44	6603	Detected	34.61	79.5	2.2E+03	1.51E+05	–
45	6524	Detected	37.98	78.9	2.4E+02	1.69E+04	–
47	6615	Detected (LOD)	36.51	79	NA	NA	Detected but not quantifiable
48	6734	Detected	28.73	78.5	9.9E+04	6.84E+06	–
49	6735	Detected	33.04	79	6.0E+03	4.17E+05	–
50	6733	Detected	31.49	79.1	1.6E+04	1.14E+06	–
51	6985	Detected (LOD)	34.04	78.2	1.69E+00	1.17E+02	Detected but not quantifiable
52	6992	Detected (LOD)	33.25	78.3	3.03E+00	2.11E+02	Detected but not quantifiable
53	7159	Detected	37.13	78.5	6.24E+02	4.33E+04	–
54	7160	Detected	37.43	78.5	5.13E+02	3.56E+04	–
55	6578	Detected	33.99	79.5	7.0E+01	4.87E+03	–
56	6576	Detected (LOD)	35.64	79.5	NA	NA	Detected but not quantifiable
60	7099	Detected (LOD)	34.28	78.3	1.41E+00	9.81E+01	Detected but not quantifiable
61	5430	Detected	32.91	78.5	2.3E+02	1.27E+04	–

*In 43 blood samples, B. miyamotoi was detected, i.e., 5.22% of the samples analyzed. For 22 samples, the detection was inferior to the limit of quantification of the B. miyamotoi PCR kit thus these samples could not be quantified. These 22 samples are said to be positive in limit of detection (LOD). For 21 samples, quantification was possible. The melting temperature of the B. miyamotoi positive control is 79°C and the tolerance range for the B. miyamotoi positive Tm is 79 ± 1.5°C. The melting temperatures of the amplicons coming from the detected samples are between 78.3 and 79.5°C. These amplicons are due to specific amplifications produced by the B. miyamotoi primers*.

### Sequencing of the Amplicons Obtained

**Figure 3:**
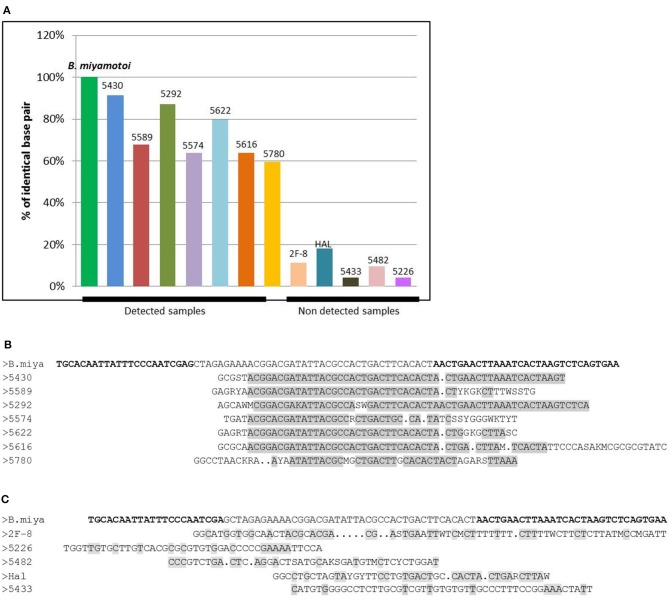
**(A)** Graph of the percentage of identical base pairs for the detected and undetected samples for *B. miyamotoi* PCR. The percentage of identical sequenced bases is >60% for the detected samples which display long runs of identical bases **(B)** and <20% for the samples not detected in PCR with no long runs of identical nucleotides **(C)**. The sequence of interest of *B. miyamotoi* is a very short sequence (94bp). When sequencing small sequences, the first bases may not be recognized by the sequencer because of the brevity of the sequence, which explains why for some positive samples, sequencing only returns 60% of the bases of the sequence.

In order to confirm the specificity of the *B. miyamotoi*amplicons in the positive samples, eight positive and five negative samples (13 in total) were selected and subjected to DNA sequencing. Negative samples were sequenced in order to confirm that the readings retrieved were specific of positive samples and not a sequencing artifact. Negative samples had <20% similarity to the expected amplified sequence with no long runs of similar nucleotides (Figure 3A), while all positive samples showed greater than 60% similarity, even up to more than 90% for some amplification results (91% for sample number), with high number of similar consecutive nucleotides (Figures 3B,C). Giving the shortness of the amplified sequence (94 bp), as for any sequencing, the beginning (and sometimes a few base pairs at the end) of the sequence is often not available (used by the primer which is not sequenced) and accounts for the fact that only 60% of some of the sequenced amplicons are read.

### Clinical Charts of Symptomatic Patients

Among the 43 patients with a positive PCR, 31 filled the questionnaire with their physician. Out of the 31 reported cases, 11 had their place of residence in Bretagne or the Loire Valley (West); the others were spread throughout the territory with a slight predominance for the South-West and the Rhône-Alpes region (South-East).

The duration of signs and symptoms divided the patients into two groups. For six patients, the duration of signs and symptoms was less than one year, while for 25 patients, signs and symptoms persisted on the long term (Table 5). Two patients have been sick for almost 30 years, two other patients for at least 20 years, the remaining patients between 1 and 19 years.

**Table 5:** Clinical signs and symptoms of 31 patients^*^ with a PCR, performed from a blood sample, positive for *Borrelia miyamotoi*.

	**Number of patients (%)**	**Description**	**Number of patients (%)**
Duration of signs and symptoms		Less than 1 year Long term^**^	6 (19.4) 25 (80.6)
**Signs and symptoms**			
Erythema migrans	5 (16.1)		
Asthenia	31 (100)	Moderate	10 (32.2)
		Strong	21 (67.8)
Joint pain (often migrating)	31 (100)	Moderate	9 (29)
		Strong	22 (71)
Neurocognitive disorders	31 (100)	Loss of concentration, attention, memory and/or speech	
Sleeping disorders	31 (100)		
Other pains		Myalgia	25 (80.6)
		Including muscle cramps	16 (51.6)
		Cephalalgia (strong)	20 (64.5)
Thermoregulation disorders and associated signs		Chilliness	18 (58)
		Hot flushes	16 (51.6)
		Sweats (mainly at night)	15 (48.4)
		Relapsing fever	11 (35)
Respiratory symptoms		Chest tightness/lack of air	13 (41.9)
		Dyspnea	6 (19.4)
Balance disorders/malaises		Repeated falls	3 (9.7)
		Repeated malaises	2 (6.5)
Visual disturbances		Amputation of the visual field	1 (3.2)
		Diplopia	1 (3.2)
Other neurologic disorders		Parsonage-Turner syndrome	2 (6.5)
		Multiple sclerosis	1 (3.2)
		Manic depressive psychosis	1 (3.2)

* *These 31 patients are those, among the 43 patients of the study, who fulfilled with their physician a questionnaire*.

** *For six patients, the duration of signs and symptoms was less than one year, while for 25 patients, average duration of signs and symptoms was 9 years, with a range from 1 to 30 years. Two patients have been sick for almost 30 years, two other patients for at least 20 years, the remaining patients between 1 and 19 years*.

The results of the Lyme borreliosis ELISA test (commercial tests, performed in city laboratories) which is based on three species of the *B. Burgdorferi s.l*. complex (*B. burgdorferi s.s*., *B. afzelli, B. garini*), are negative for 19 patients (76% of 26 informed cases), doubtful in three cases, positive in three cases, and not informed in six cases. Western-blot was negative in nine cases (50% of 18 informed cases), positive in nine cases (including three formerly positive and one doubtful with previous ELISA test). For 13 patients, Western-blot was not performed (eight cases) or no result was informed (five cases).

Erythema migrans, a sign specific for Lyme disease, was not frequent (Table 5).

Other recorded clinical signs and symptoms are reported in Table 5. Asthenia was constant and was usually happening quite abruptly, corresponding to a change of life for patients, in their personal, professional and sport activities. The asthenia intensity was graded with a 0–5 scale, and reported as “moderate” (score of 1–3) or “strong” (score of 4 or 5). The cephalalgia intensity was graded with a 0–5 scale, and reported as “moderate” (score of 1–3) or “strong” (score of 4 or 5). Some patients with neurocognitive disorders were unable to answer questions correctly. In these cases, it is their family members or relatives who answered for them.

A significant proportion of patients experienced signs suggesting thermoregulation disorders, including episodes of relapsing fever, an interesting fact since *B. miyamotoi* belongs to a group responsible for relapsing fever.

## Discussion

*B. miyamotoi* belongs to the relapsing fever group of pathogenic *Borrelia*. Rather few cases of *B. miyamotoi* infection were identified in humans. There is a debate about the clinical picture of the disease. It can be responsible for relapsing fever; however some clinical cases were more similar to Lyme borreliosis, including some cases with erythema migrans. The present study, conducted in France, is the largest case series of *B. miyamotoi* infection detected in patients suffering from long term persistent syndrome. It complements the Russian study by Karan et al. published in 2018 (28), which was conducted at the early stage on patients presenting with acute symptoms after a tick bite. The results of both studies suggest that *B. miyamotoi* is more frequent in humans than previously thought. We provide a gross description of the clinical signs and symptoms and the duration of the disease. However the lack of power of the clinical part of the study does not allow a definite conclusion about a precise clinical description. *B. miyamotoi* has a particular position in the genus *Borrelia*. No serology is available in routine. The sensitivity of PCR for the species belonging to the *B. burfdorferi s.l*. complex appears to be rather low, especially in blood. The sensitivity of PCR for *B. miyamotoi* is not known. The species *B. miyamotoi* may also suffer from a deficit of detection. PCR may become a useful means for the detection of *Borrelia*, amplifying the *fla* gene for flagellin, specific of *Borrelia* species. The *fla* gene is present in all *Borrelia* species with several conserved portions between the different *Borrelia species*. The choice of the sequence of interest should depend on the chosen target i.e., *B. burgdorferi sensu stricto* or *sensu lato*. As the *fla* gene is also present in the genome of *B. miyamotoi*, it is possible that *B. miyamotoi* could have been detected and included in the *B. Burgdorferi s.l*. complex. The use of a kit specific for *B. miyamotoi* target probably favored our isolation. *B. miyamotoi* is a species apart, pathogenic and probably non-commensal as suggested by the fact that the healthy students of the University of Angers are not infected, while being in a rural area rich in ticks.

**Figure 4:**
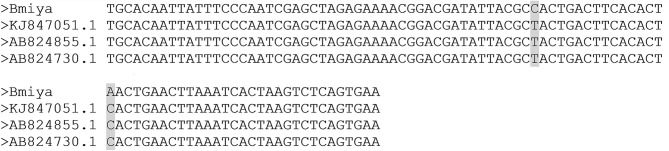
Alignment of the *Borrelia miyamotoi* French strain with the other European *Borrelia miyamotoi* strains (KJ847051.1, AB824855.1, AB824730.1). The sequences show a single nucleotide difference that does not affect the PCR and the PCR efficiency.

As evidenced in the publication by Reiter et al. (30) and the sequence alignment, the sequence fragment used for the detection of *B. miyamotoi* in the blood of the French patients tested is strongly homologous to other European strains of *B. miyamotoi* found in patients (KJ847051.1, AB824855.1, AB824730.1), showing only two nucleotides differences between sequences; differences which does not affect detection by PCR (see Figure 4). The *glpQ* gene was chosen for its specificity as it is, to the best of our current knowledge, only present in *B. miyamotoi* strains. Thus, the detection of a said gene is indicative of the presence of the pathogen. Additional genes often show lack of specificity, especially the 16S RNA or *flaB* genes, which are highly conserved in all *Borrelia* species, including those of the relapsing fever group. Indeed, sequencing of really short PCR fragments is often challenging as the first 20 or so nucleotides (the primer) are already “lost” due to the intrinsic nature of sequencing which does not “read” the primer used. Our claim is not with the percentage of similarity *per se*, our claim is in the consecutiveness of those homologous nucleotides. Forty identical consecutive bases, even in a 94 bases long fragment, deriving from a gene is specific of a pathogen.

The sequencing carried out shows that the amplicon obtained by PCR corresponds, for more than 60% and up to 90% of the purine and pyrimidine bases, to the desired target sequence specific for the *B. miyamotoi* species.

During the study period, *B. miyamotoi* was found with a high frequency (5.22%) compared to the other *Borrelia* species, i.e., *B. burgdorferi s.l*. (including *B. burgdorferi s.s*. *, B. garinii, B*. *afzelli, B. bissettii, B. spielmani, B. kurtenbachi*): 0.73% and *B. hermsii*: 0.36% (data not shown).

This pilot study, conducted in patients from various regions in France, suggests that *B. miyamotoi* infection could be more frequent in humans than previously thought and perhaps more frequent than other species of *Borrelia*, especially those classically responsible for Lyme disease. The signs and symptoms of persistent polymorphic syndrome possibly due to a tick bite are close to those described as post-treatment Lyme disease syndrome. Erythema migrans was observed in 16.1% of the patients, but data are insufficient to rule out a previous infection with *B. burgdorferi s.l*. However the responsibility of *B. miyamotoi* in some cases of erythema migrans is probable since, in the study looking at the early stage of the tick-borne infection, 3% of patients with an erythema migrans had a positive blood PCR for *B. miyamotoi* (28). Our data suggest that the disease may be persistent, even on the long term and that this species of *Borrelia* may not cross-react with *B. burgdorferi* serology. Asthenia, joint pain, neurocognitive disorders and sleep disorders were reported by all patients. Episodes of relapsing fever were observed in 35.5% of the cases. A large prospective study is needed to further describe this infection in well-defined populations.

In conclusion, among French patients suffering from a persistent polymorphic syndrome possibly due to a tick bite (SPPT), a syndrome close to post-treatment Lyme disease syndrome (PTLDS), 43 out of 824 (5.22%) had *B. miyamotoi*in their blood identified by specific real-time PCR, including 22 cases at the detection limit and 21 quantifiable cases. This is the first detection of this bacterial species in humans in France. Sequencing showed the specificity of the detected DNA as *B. miyamotoi*. This study highlights that the lack of detection of *B. miyamotoi* is not due to the absence of this particular species of *Borrelia* in France, but rather because this species was not sought out. Clinical studies designed to evaluate the correlation of PCR results with clinical signs and symptoms should be done to better investigate patients suffering from persistent polymorphic signs and symptoms of unclear origin.

## Data Availability Statement

The raw data supporting the conclusions of this article will be made available by the authors, without undue reservation, to any qualified researcher.

## Ethics Statement

The human studies were reviewed and approved by the Comité de protection des personnes CPP SUD 9EST VI Clermont Ferrand, France. All patients and control persons provided written informed consent.

## Author Contributions

All authors listed have made a substantial, direct and intellectual contribution to the work, and approved it for publication.

### Conflict of Interest

MF is CEO of ADNucleis. The remaining authors declare that the research was conducted in the absence of any commercial or financial relationships that could be construed as a potential conflict of interest.
